# Prefabrication of an Axially Vascularized Bone Graft: Proof of Concept for a BMP-2-Based Cell-Free Approach

**DOI:** 10.3390/jfb17100520

**Published:** 2026-10-15

**Authors:** Pablo Pfister, Gangyu Zhang, Sébastien Pigeot, Adrien Moya, Alexander Haumer, Robert Paillaud, Dirk J. Schaefer, Tarek Ismail, Ivan Martin, Arnaud Scherberich

**Affiliations:** 1 Department of Plastic, Reconstructive, Aesthetic, and Hand Surgery, University Hospital Basel, 4031 Basel, Switzerland; 2 Department of Biomedicine, University Hospital Basel, University of Basel, 4051 Basel, Switzerlandrobert.paillaud@unibas.ch (R.P.); ivan.martin@usb.ch (I.M.)

**Keywords:** bone tissue engineering, BMP-2, bone morphogenetic protein 2, bone regeneration, microsurgery, reconstructive surgery

## Abstract

Reconstruction of large craniofacial bone defects typically relies on vascularized autografts, but involves donor-site morbidity and limited availability. Tissue-engineered vascularized grafts could address this gap; however, current strategies remain limited by in vitro cell manipulation, regulatory complexity, high costs, and difficult clinical scaling up. We previously prefabricated a maxillary graft combining a devitalized bone matrix (DBM), freshly isolated human adipose-derived cells, known as the stromal vascular fraction (SVF), and Bone Morphogenetic protein-2 (BMP-2). Here, we tested if cells were essential by developing a cell-free approach with only DBM (SmartBone) and BMP-2. SmartBone blocks were loaded with BMP-2 (60 µg/mL in a fibrin hydrogel) with or without SVF cells and implanted subcutaneously in nude mice for 12 weeks (ectopic model) or combined to an axial arteriovenous (AV) bundle in nude rats (785 mm^3^) for 2 or 12 weeks (vascularized model). Bone formation was assessed by X-ray tomography and histology. Across both mice and rats, BMP-2 alone was sufficient to generate robust and organized bone, and adding SVF cells provided no additional benefit at this BMP-2 dosage. In mice, BMP-2 grafts formed lamellar bone with marrow, while grafts without BMP-2 produced only calcified fibrous tissue. Bone and marrow volumes did not differ between BMP-2 and BMP-2 + SVF groups (*p* > 0.05). In rats, both conditions showed significant increases in mineralized volume from 2 to 12 weeks (*p* < 0.05). In conclusion, the dose of BMP-2 tested here, combined with DBM, is enough to robustly generate pedicled bone grafts while using off-the-shelf, approved materials and microsurgical prefabrication, providing a simplified, translationally feasible approach for the reconstruction of large bone defects.

## 1. Introduction

Reconstructing large bone defects in the maxillofacial region remains a significant clinical challenge, typically requiring autologous vascularized bone grafts [1]. This reconstructive option provides a stable foundation for supporting effective masticatory rehabilitation, either with removable or implant-supported prostheses [2]. However, autologous bone grafting is limited by restricted availability, significant donor site morbidity, and, in some cases, the inability to achieve functional oral rehabilitation [3].

To address these limitations, cell-based bone tissue engineering (BTE) has emerged as a promising approach. Numerous animal studies have demonstrated BTE’s capacity to generate bone in both non-bony sites (ectopic bone formation) and bony sites (orthotopic bone formation) [4,5,6,7,8,9]. In addition to not always being effective in preclinical models, BTE approaches remain constrained by the difficulty of scaling up from animal models to human size applications [4].

We have previously demonstrated the feasibility of a BTE approach, by ectopically prefabricating an axially vascularized bone graft in a rat model [10,11]. This approach utilized an arteriovenous (AV) bundle, generating a pedicled bone graft substitute with intrinsic vascularization, as opposed to relying solely on vascular ingrowth from surrounding tissues. In two prior preclinical studies, we combined an AV bundle model with either adipose-derived stromal vascular fraction (SVF) cells pre-cultured under perfusion on large hydroxyapatite porous sponges [10,11], or bone-marrow-derived stromal cells differentiated into hypertrophic cartilage to induce endochondral ossification [10,11]. Although effective in terms of bone formation and vascularization, those approaches require an in vitro cell culture step prior to implantation and fall under the regulation of advanced therapy medicinal products (ATMPs). Therefore, their translational potential is more limited due to high costs, complex logistics and challenging regulatory requirements to guarantee reproducible quality and safety under Good Manufacturing Procedures (GMP) conditions.

In a first-in-human case report, we used intraoperatively collected, non-expanded cells and applied the concept of the AV-bundle to prefabricate an axially vascularized bone graft [1]. In particular, for maxillary reconstruction in a 39-year-old patient following a five-year tumor-free interval, we implanted a devitalized bone matrix (DBM) of human origin seeded with freshly isolated, autologous SVF cells, combined with bone morphogenetic protein-2 (BMP-2) embedded in a fibrin hydrogel. This protocol builds on prior findings demonstrating that BMP-2 can induce ectopic bone regeneration in non-skeletal sites, thereby enhancing the osteogenic potential of the engineered graft [12,13,14,15]. The construct was assembled and combined to the serratus branch of the thoracodorsal artery and vein of the patient for a nine-month prefabrication phase. Although rapid vascularization and appearance of bone-like metabolic activity was observed within few weeks, substantial graft resorption gradually occurred during the prefabrication phase, leaving only 9% of bone tissue at the time of subsequent transplantation after 8 months. To partially compensate for the resorption, additional bone was harvested from the patient’s tabula externa and combined with the prefabricated bone graft. Thereby, we successfully reconstructed the maxillary defect with the prefabricated flap, achieving restoration of midfacial symmetry, separation of the nasal and oropharyngeal cavities, support for physiological swallowing and restoration of mastication function.

The aim of the present study was to further develop the previously established prefabricated, axially vascularized engineered bone grafts in order (i) to reduce the observed massive resorption of the graft and (ii) to avoid—if possible—the use of living autologous cells. We thus decided to replace the DBM of human origin by SmartBone, a bovine-derived DBM coated with bioresorbable polymers and collagen fragments, previously reported to resorb very slowly as compared to human DBM and maintain for at least 1–2 years upon implantation into patients [16]. This new bovine DBM was tested as a preclinical validation in this study. In addition, we investigated the specific contribution of SVF cells delivered in addition to BMP-2.

## 2. Methods

### 2.1. SVF Cell Isolation

Human adipose samples, in the form of liposuction samples or minced excision material of human adipose tissue, were obtained under the general hospital consent (University Hospital of Basel, Basel, Switzerland). Following informed consent from the patient, samples were anonymized in accordance with the Ethical Committee of Northwest and Central Switzerland (EKNZ, Request BASEC #Req-2026-00138) and following Article 2.2.c of the Swiss Human Research Act (HRA, RS 810.30). SVF cells were obtained from 4 healthy female donors (age 45–69) and isolated by enzymatic digestion with Collagenase Type II, followed by several centrifugation and purification steps as previously described [17]. Nucleated cells in SVF were counted with a LUNA-FX7 cell counter after staining with acridine orange/propidium iodide (all from Logos Biosystems Inc., Anyang-si, Republic of Korea).

### 2.2. Graft Seeding and Preparation

Cylinders and blocks of a bovine DBM product (SmartBone, IBI SA, Mezzovico-Vira, Switzerland) were used. The characteristics of SmartBone regarding resorbability, mechanical properties and microstructure have been previously demonstrated [18,19,20]. The dimensions of the cylinders used in the AV bundle rat model were obtained by assembling two centrally pre-drilled blocks, each with a diameter of 10 mm and a height of 5 mm. The blocks used in the mouse subcutaneous model were 5 mm × 5 mm × 3 mm in size. Prior to use, the bone grafts were pre-wetted with phosphate-buffered saline (Sigma-Aldrich, St. Louis, MO, USA) for 5 min.

During the seeding process, the blocks were placed on silicone molds to prevent significant outflow of seeding fluid from the SmartBone scaffolds. The seeding step began with the application of 150 µL of hydrogel of the fibrinogen component of a fibrin hydrogel (Tisseel, Baxter, Deerfield, IL, USA) per cm^3^ of DBM, delivered with a pipette tip inserted into the pre-drilled hole. The precise composition of the fibrinogen component varied depending on the experimental group: in specific groups, BMP-2 was added or not at a concentration of 60 µg/mL of fibrin gel, while SVF cells were included at concentrations of 0, 1 × 10^6^, 4 × 10^6^, or 10 × 10^6^ cells/cm^3^.

The fibrinogen component was left on the scaffolds for 5 min before the thrombin component of the fibrin gel was added at a volume of 150 µL/cm^3^ DBM.

### 2.3. Implantation Phase: Mouse Model

After the seeding process, the blocks (maximum 4 per mouse) were implanted subcutaneously in athymic CD1 nu/nu female nude mice (Charles River Laboratories, Freiburg, Germany). Mice were operated under the permission of the Federal Veterinary Office (permit BS 1797) as previously described [21]. Animals were monitored thereafter according to the animal permit guidelines. Experiments were performed in accordance with the ARRIVE guidelines, as requested and enforced by the local Veterinary Office. Euthanasia was performed by CO_2_ following the protocol defined by the University of Basel to ensure a humane sacrifice of the mice.

### 2.4. Implantation Phase: Rat Model

Animal procedures were approved by the Federal Veterinary Office (permit BS 2598). Before surgery, nude rats (Charles River Laboratories, Germany) were injected subcutaneously with 0.05 mg/kg of buprenorphine (Temgesic) or extended-release buprenorphin 0.65 mg/kg (Ethiqa XR). Anesthesia was induced and sustained with 2.5% Isoflurane in 1 L/min O_2_. The rats were put in a supine position, placed on an electrically heated mat to maintain body temperature, disinfected and a sterile operating field was established. Through an incision approximately 15 mm in length along the left groin, the superficial inferior epigastric artery and vein were dissected free of most surrounding tissue. Upon distal ligation, the created AV bundle was inserted through a 1.25 mm axial pre-drilled hole in the center of the graft. A subcutaneous pouch was created by blunt dissection and the graft was carefully inserted. In order to prevent any ingrowth of vessels from surrounding tissues, grafts were wrapped in a semipermeable inorganic-based silicone membrane (Biobrane, UDL Laboratories Inc., Rockford, IL, USA). To prevent the rats from self-trauma of the suture line, subdermal and intradermal sutures with absorbable material were applied (5-0 Monocryl thread from Ethicon, Raritan, NJ, USA). The rats recovered under infrared light and were transferred back to their cages with free access to food and water. Animals were monitored thereafter according to the animal permit guidelines. Experiments were performed in accordance with the ARRIVE guidelines, as requested and enforced by the local Veterinary Office. Euthanasia was performed by CO_2_ following the protocol defined by the University of Basel to ensure a humane sacrifice of the rats.

### 2.5. Microtomography

At the end of the in vivo period, animals were sacrificed and samples were retrieved and fixed overnight in 4% formalin (Formafix, Hittnau, Switzerland). Then, microcomputed X-ray tomography (μCT) data were acquired by using a high-resolution scanner (SkyScan1172, Skyscan, Kontich, Belgium) and 0.5 mm aluminum filtered X-rays (applied voltage 50 kV; current, 200 μA). Transmission images were acquired during a 360° scan rotation with an incremental rotation step size of 0.25°. Reconstruction was performed using a modified Feldkamp algorithm at an isotropic voxel size of 4–10 μm. Three-dimensional (3D) rendering, thresholding, segmentation, and 3D measurements were performed using VG Studio MAX 2.2 software (Volume Graphics, Heidelberg, Germany).

### 2.6. Histology

After X-ray tomography and slow decalcification with EDTA, samples were embedded in paraffin and sectioned at different levels in 5 μm thick sections. Standard histological haematoxilin and eosin (H&E), Masson’s trichrome (Réactifs RAL, Martillac, France) and Movat’s pentachrome (Statlab, McKinney TX, USA) were used. Immunohistochemistry was performed with primary antibodies against human nuclei (MAB4383, Sigma-Aldrick, St. Louis, MO, USA), Human Leukocyte Antigen 1 (ab70328, Cambridge, UK). HLA-1-specific staining was visualized with the Vectastain ABC kit and the Vectastain Fast Red kit (Vector laboratories, Burlingame, CA, USA) according to the manufacturer’s protocols. The human-nucleus-specific staining was performed with a Ventana Discovery Ultra (Roche Diagnostics, Rotkreuz, Switzerland) automated slide stainer. In brief, tissue sections were deparaffinized and rehydrated. Antigens were retrieved by a protease (Protease 3, ref. 760-2020, Roche Diagnostics) digestion for 20–44 min at 37 °C. The primary antibody was manually applied and incubated for 1 h at 37 °C. After washing, the secondary antibody was incubated for 1 h at 37 °C. The detection step was performed with the Ventana DISCOVERY ChromoMap DAB (ref. 760-159 Ventana) detection kit. Afterward, the slides were counterstained with hematoxylin II, followed by a bluing reagent (respectively, Cat. no. 790-2208 and 760-2037, Roche Diagnostics). Sections were then dehydrated, cleared, and mounted with permanent mounting and coverslips.

Images of the histological sections were acquired with a Nikon Ti2 widefield microscope, a Nikon DS-Ri2 camera (Nikon, Tokyo, Japan) and a CFI Plan Apo Lambda NA 0.75×, 20× objective (Nikon, Tokyo, Japan). The software used was the NIS- Elements AR 5.21.03 (Nikon, Tokyo, Japan).

### 2.7. Quantifications

H&E-stained sections were used to quantify bone formation. Bone was identified by autofluorescence and manual thresholding. Tissue was categorized into two classes, namely bone and bone marrow, by using the open-source software QuPath v0.3.2.41. Annotations for each class were manually drawn.

### 2.8. Statistical Analysis

All results are expressed as mean values with standard deviations. ANOVA analysis and *t*-tests were performed at a level of statistical significance *p* < 0.05 by using the Prism software (GraphPad Software Inc., San Diego, CA, USA).

For the mouse experiment, 32 grafts were implanted in 8 mice, with four grafts per mouse. Histomorphometric measurements were analyzed at the graft level; therefore, the reported *n* represents the number of grafts analyzed. For comparisons between two groups, an unpaired two-tailed Student’s *t*-test was used. For comparisons involving more than two groups, one-way ANOVA was performed. For the rat experiment, comparisons between two groups were likewise performed using an unpaired two-tailed Student’s *t*-test, whereas ANOVA was used for comparisons involving more than two groups. Statistical significance was defined as *p* < 0.05. Exact sample sizes for each analysis are provided in the respective figure legends.

For the rat experiments, SVF cells were incorporated at three different concentrations (1 × 10^6^, 4 × 10^6^, or 10 × 10^6^ cells/cm^3^). For statistical analysis, these three SVF concentrations were combined and analyzed as a single SVF-positive experimental group. Accordingly, the SVF-positive groups reported in the Results represent the pooled 1 × 10^6^, 4 × 10^6^, and 10 × 10^6^ cells/cm^3^ conditions.

## 3. Results

### 3.1. Mouse Model: Bone Tissue Formation

In the subcutaneous mouse model, a total of 32 grafts, based on SVF cells and/or BMP-2, were implanted in eight mice (Figure 1A) for 12 weeks. One animal lost a significant amount of body weight during the first postoperative week and was therefore sacrificed in accordance with the animal permit. This resulted in a total of 28 grafts available for analysis.

The thresholding of µCT data for densities corresponding to de novo mineralization, achieved by visually controlling the selected voxels, revealed mineralized areas, distinguishable from the devitalized bone-based scaffolding materials. They were observed in all four experimental conditions (red arrows, Figure 1B), even when only DBM (i.e., without SVF cells and/or BMP-2) was implanted. However, histological analyses of the mineralized tissue areas on H&E-stained sections showed that they corresponded to two different types of tissue, namely areas of new bone tissue formation, with lamellar bone and bone marrow, in the conditions with BMP-2 and SVF + BMP-2 (red arrows, Figure 1C) and areas of calcified fibrous tissue for negative control (materials alone) and SVF cells only (yellow arrows, Figure 1C). The quantification of newly formed bone by segmentation of H&E-stained samples showed no significant difference in bone area between the BMP-2 (0.28 ± 0.25 × 10^6^ µm^2^) and SVF + BMP-2 (0.17 ± 0.14 × 10^6^ µm^2^) groups (*p* > 0.05, Figure 1D). Similarly, the quantification of newly formed bone marrow pockets showed no significant difference between the BMP-2 (0.33 ± 0.37 × 10^6^ µm^3^) and SVF + BMP-2 (0.16 ± 0.23 × 10^6^ µm^3^) groups (*p* > 0.05, Figure 1E). In this animal model, BMP-2 was found to be the determinant factor in bone and bone marrow formation. In addition, our data showed that SVF cells associated with SmartBone were not able to induce bone formation.

### 3.2. Rat Model: Bone Tissue Formation and Bone Prefabrication

In the rat model, a total of 22 grafts, based on BMP-2-loaded grafts, with or without SVF cells, were implanted in 22 animals (Figure 2A) for 2 or 12 weeks. Four animals had to be sacrificed due to self-inflicted wound openings and graft removal, in accordance with the animal permit. Additionally, one animal developed a postoperative hematoma with a non-viable arteriovenous bundle upon explantation and was excluded from analysis. This resulted in a total of 17 grafts available for analysis (nine grafts at the 2-week and eight grafts at the 12-week timepoint). Samples were fixed with formalin and subjected to analysis and quantification by tridimensional X-ray microtomography. Newly formed mineralized tissue was distinguishable from the scaffolding material (visible already at 0 weeks, prior to implantation) and became denser over time (Figure 2B) in both experimental conditions. The mineralization, however, could not be segmented between the scaffolding material and the newly formed bone. The MV/TV values therefore represent the total mineralized tissue. A quantitative volumetric analysis combining both experimental groups revealed a significant increase in the proportion of mineralized volume between the 2-week (4.74% ± 0.01) and 12-week (6.50% ± 0.01) time points (*p* = 0.009, Figure 2C). Similar to the non-vascularized ectopic mouse model, the SVF and No-SVF groups showed no significant difference in terms of total mineralized volume in the AV-bundle-based model, neither after two weeks (4.82% ± 1.1 vs. 4.43% ± 0.01, *p* > 0.05, Figure 2C) nor after 12 weeks (6.79% ± 0.02 vs. 6.40% ± 0.01, *p* > 0.05, Figure 2C). Histological analysis showed de novo bone formation in both experimental groups and in 14 out of 17 constructs. The three constructs showing no new bone formation were all from the SVF-cell-based group. Sections stained with H&E and Movat’s pentachrome (Figure 2D) exhibited areas of new bone formation already after two weeks of implantation (red arrows, Figure 2D). The bone morphology thereafter showed increased maturation at 12 weeks (Figure 2D). Bone formation was found only in the vicinity of the central AV bundle at two weeks but extended all over the material up to the rim after 12 weeks. Quantification of the newly formed bone area per section, by segmentation of H&E pictures, revealed a significant (combination of both experimental groups) nearly fourfold increase between the 2-week (0.23 ± 0.26 × 10^6^ µm^2^) and 12-week (0.98 ± 0.92 × 10^6^ µm^2^) time points (*p* < 0.05, Figure 2E). Consistent with the data in the mouse model, no significant difference was observed between the SVF and No-SVF groups at two weeks (0.18 ± 0.19 vs. 0.39 ± 0.50 × 10^6^ µm^2^, *p* > 0.05, Figure 2F) and 12 weeks (0.98 ± 0.93 vs. 1.00 ± 1.24 × 10^6^ µm^2^, *p* > 0.05, Figure 2G).

Finally, when comparing the mean of the density of de novo bone formation between the two animal models, no statistical difference was found (0.90 ± 0.83 (mouse) vs. 1.25 ± 1.17 (rat) × 10^4^ µm^2^, *p* > 0.05, Supplementary Figure S1).

### 3.3. Description of Ossification and Contribution of Human Cells in the Rat Model

Masson’s Trichrome staining of tissue sections revealed areas containing chondroblasts and chondrocytes, suggesting an endochondral ossification pathway (Figure 3A, yellow arrows). These regions also exhibited early transition to bone formation (Figure 3A, red arrows), in both experimental conditions. After 12 weeks of implantation the bone morphology showed dense collagen and mineralized bone with areas of more mature bone stained in red (Figure 3B, blue arrows) in both experimental conditions.

Next, we aimed to identify the contribution of human cells to the newly formed bone at 12 weeks. To this end, we first characterized the newly formed bone by using the fluorescent properties of its collagen content, captured on the GFP channel. Both the newly formed bone (Figure 3C,D, white arrows) and the SmartBone scaffold made of bovine DBM (Figure 3C,D, asterisks) exhibited fluorescent signals, but were distinguishable. Using a specific anti-human nucleus antibody, we found no positive cells in any of the 12-week sections, neither in areas of mature bone formation (Figure 3D, white arrows) nor in the surrounding connective tissue.

In one sample from the 2-week time point, we were able to identify human-nucleus-positive cells in the connective tissue, but never in the newly formed bone areas (Supplementary Figure S2).

## 4. Discussion

The present study provides compelling preclinical evidence that a cell-free strategy using a clinically approved bone substitute supplemented with BMP-2 alone can generate mature, vascularized bone structures in both ectopic and axially vascularized prefabrication animal models. The data confirm the potent osteoinductive capacity of the dosage of BMP-2 used in our experiments, which, without cellular supplementation, led to formation of organized lamellar bone, including morphological evidence of hematopoietic marrow. This effect was consistently observed across both subcutaneous implantation in mice and the more clinically relevant AV-bundle model in rats.

In the mouse model, we confirmed that BMP-2 is a critical inducer for the formation of mature bone and bone marrow over a 12-week period. Interestingly, the addition of SVF cells did not result in enhanced bone formation, as no significant differences were observed between the BMP-2 groups with or without SVF. Furthermore, SmartBone alone, irrespective of SVF cell presence, exhibited only localized calcifications on µCT and histological analysis, without evidence of new bone or marrow formation. These findings confirm the notion that a devitalized bone scaffold cannot induce ectopic bone formation in the absence of an additional osteoinductive stimulus, such as BMP-2.

Following the results of the subcutaneous implantation model, we aimed to scale up the graft and introduced the application of the AV bundle for the two conditions that had previously demonstrated bone formation: BMP-2 alone and BMP-2 combined with SVF cells. Consistent with the mouse model, both conditions resulted in bone formation after a 2- and 12-week implantation period. Despite the graft cross-sectional area being approximately three times larger in the rat model and spatially isolated from surrounding tissue by a semipermeable membrane, the relative extent of bone formation remained comparable between the two models, while the absolute bone volume was substantially greater in the AV-bundle constructs. This is relevant, since one major challenge in translational BTE approaches is the lack of sufficient vascular supply in large size constructs, potentially resulting in hypoxic damage [4]. Our approach provides a large central vessel into the construct, able to bypass this critical challenge. The AV-bundle configuration is particularly significant, as axial vascularization is essential for the long-term survival and functional integration of large bone grafts in reconstructive surgery [22]. By demonstrating that BMP-2 alone can induce bone formation along a surgically introduced vascular axis, this work advances the feasibility of translating axially vascularized, prefabricated grafts into a clinical setting. We have previously shown that the inset of an AV bunde leads to a robust vascularisation pattern, but in our experimental setup we did not specifically investigate the effect of the AV bundle addition in regard to bone-forming capacity, as no negative control without an AV bundle was used [10,11].

In this experimental setting, the addition of SVF cells did not produce a statistically significant enhancement of bone formation in either animal model. To evaluate the presence of human cells in the AV-bundle model, we employed a specific anti-human-nucleus antibody. At the 2-week time point, human-nucleus-positive cells were detected in connective tissue in one sample, whereas no human-nucleus-positive cells were identified within newly formed bone. At 12 weeks, no human-nucleus-positive cells were detected in the analyzed sections. These findings indicate that human cells were not detectable in mature bone at the terminal time point, but they do not exclude transient survival, early recruitment, or paracrine effects during the initial stages of regeneration. Molecular analyses and additional early time points would be required to investigate these mechanisms.

Despite evidence supporting beneficial roles of SVF cells in some bone tissue-engineering settings, we did not detect an additional bone-forming effect of SVF cells under the conditions tested here. This contrasts with our earlier work, where SVF cells were necessary to induce ectopic bone formation in a subcutaneous implantation mouse model [12]. A relevant difference is the amount of BMP-2 applied (60 µg/mL in the present study compared with 4.2 µg/mL in Mehrkens et al. [12]). The present data are therefore compatible with but do not prove the hypothesis that the high BMP-2 concentration may have masked a potential contribution of SVF cells. Other explanations, including limited cell survival or distribution within the scaffold, cannot be excluded. This possibility is consistent with previous reports showing substantial loss of implanted mesenchymal stem cells when seeded into scaffolds and placed subcutaneously [23,24,25]. Delayed SVF-cell application 2–3 weeks after implantation has been proposed to allow new blood vessels to form throughout the construct, thereby improving the local supply of oxygen and nutrients for implanted cells [4]. Importantly, the present study did not evaluate early cell survival quantitatively, BMP-2 release kinetics, or the effect of lower BMP-2 doses. These parameters should be investigated before concluding that a cell-free approach is preferable across applications. From a translational perspective, omitting a cellular component could simplify tissue procurement and processing and reduce regulatory complexity, but these potential advantages must be weighed against the biological uncertainty and safety considerations associated with BMP-2 dosing and the lack of direct evidence that the tested construct can provide clinically meaningful bone volume. The target patient population, namely individuals with head and neck cancer, may also present with nutritional deficiencies and limited fat reserves, making SVF cell harvesting challenging [26].

Clinically, BMP-2 is approved for treating spinal fusions, proximal tibial fractures and maxillary sinus augmentation [27,28,29,30]. Despite its efficacy, its use has been associated with controversy due to reported side effects ranging from wound complications to potentially life-threatening inflammatory responses, particularly when used in cervical spinal fusion procedures [31]. In addition, preclinical studies have suggested a potential link between BMP-2 and carcinogenicity, with conflicting findings showing both dose-dependent tumor suppression and enhancement [32]. The largest clinical study to date, analyzing 467,916 Medicare patients who underwent spinal arthrodesis with or without BMP-2, found no positive correlation between BMP-2 use and cancer development over a mean follow-up of three years [33]. However, it is important to note that BMP-2 dosages used in this study were not reported. A dose-dependent cancer risk could explain the variability in the literature, as much higher doses, such as 40 mg, have been associated with tumorigenic effects [34,35]. In our clinical case, we used a BMP-2 concentration of 60 µg/mL, corresponding to a total amount of approximately 0.5 mg. However, the substantially higher effective exposure used in the present rodent experiments, together with the known species-dependent responses to BMP-2, precludes direct extrapolation of these findings to humans. Furthermore, the present experiments were not designed as safety studies. Consequently, the current data should not be interpreted as demonstrating the safety or defining a clinically appropriate dose of BMP-2 for axially vascularized bone-graft prefabrication.

## 5. Limitations

Several limitations should be considered when interpreting these findings. First, the rat study included only BMP-2-containing constructs with or without SVF cells and therefore lacked DBM-only, DBM + SVF, and non-vascularized controls. The contribution of BMP-2, scaffold architecture, and the AV-bundle itself therefore cannot be separated. In particular, canalization of the scaffold may have increased the effective surface area available for host–cell colonization independently of any vascularization effect. Second, attrition in the rat model reduced the evaluable sample size from 22 to 17 constructs, resulting in smaller subgroup sizes and more limited statistical power. Therefore, the absence of statistically significant differences should not be interpreted as evidence that SVF cells have no biological effect. In the mouse model, multiple grafts originated from the same animal, meaning that the observations were not fully independent. This represents a limitation of both the experimental design and the statistical analysis and should be considered when interpreting the findings. Third, the absence of human nuclei-positive cells at 12 weeks does not exclude early cell survival, transient participation, or paracrine effects. Fourth, the histological observation of cartilaginous tissue is compatible with endochondral ossification but does not establish this mechanism. We did not perform immunohistochemical or molecular analyses for markers such as type II collagen, RUNX2, COL1A1, or osteocalcin. Finally, the quantitative histological analysis was based on sampled sections and may be subject to sampling bias. Collectively, these limitations support interpreting the study as an exploratory proof of concept rather than definitive evidence of clinical efficacy or of a negligible contribution of vascularization or SVF cells.

## 6. Conclusions

In summary, our study demonstrates the feasibility of a cell-free pedicled bone tissue engineering strategy using a bovine DBM, BMP-2, and microsurgical vascular prefabrication via an AV bundle. While BMP-2 alone was sufficient to induce robust bone formation, the addition of SVF cells did not yield a synergistic effect under the given conditions. These findings support a simplified, translationally realistic approach, minimizing regulatory hurdles and logistical complexities associated with cell-based therapies.

## Figures and Tables

**Figure 1:**
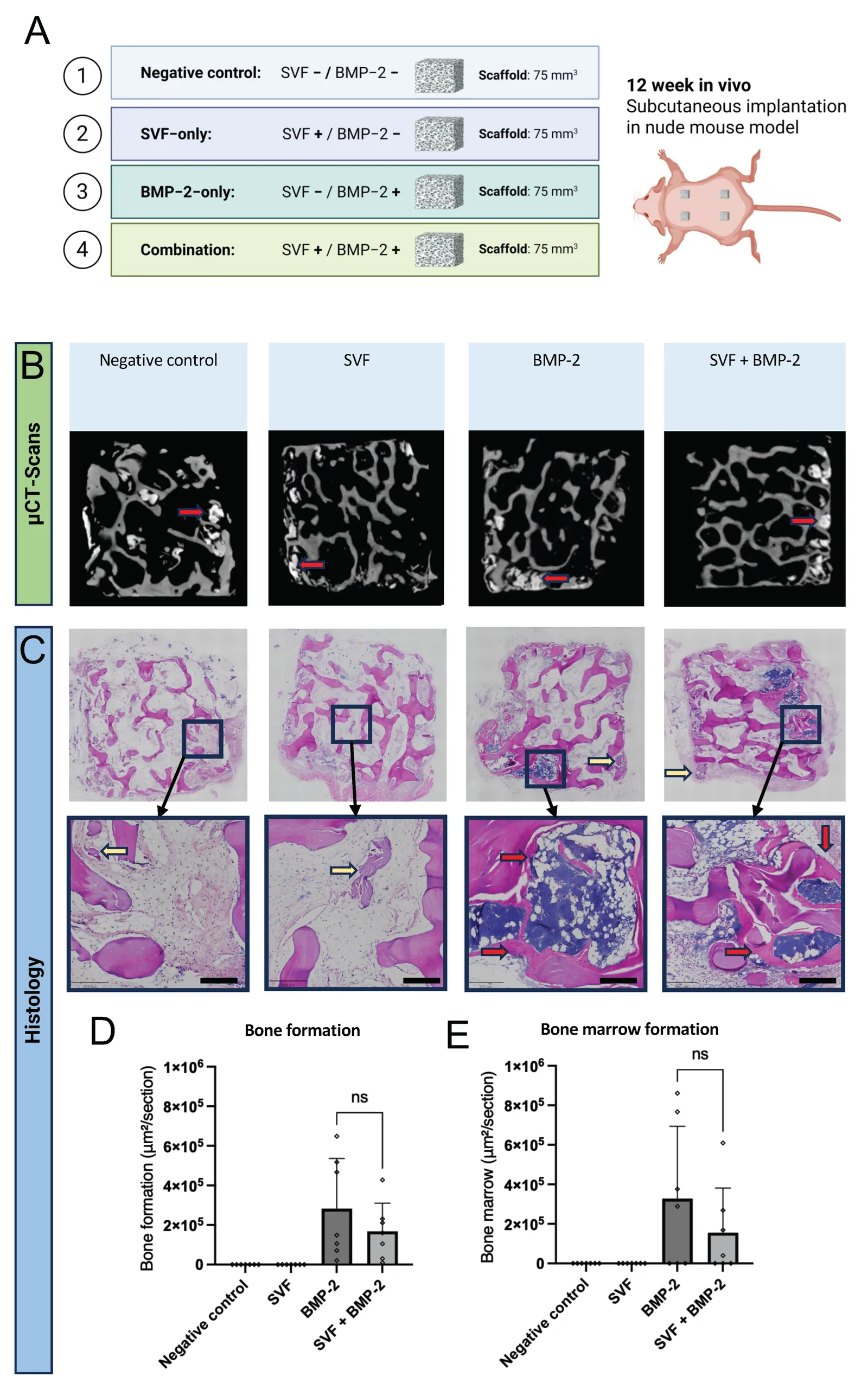
Bone formation in a nude mouse model at 12 weeks. A total of 32 grafts were implanted in 8 mice, with 4 grafts per mouse. Effect of SVF cells and BMP-2 on ectopic bone formation by Smartbone blocks after 12 weeks. (**A**) Schematic of the experimental conditions. (**B**) X-ray tomographic pictures of the samples after explantation. Red arrows show newly formed mineralized tissues. Section of smartbone (appearing in gray) is 5 by 5 mm. (**C**) H&E staining of histological sections and insets showing higher magnification (scale bar = 200 μm). Yellow arrows show mineralized fibrous tissue and red arrows show bone tissue. (**D**,**E**) Histomorphometric quantification of the section surface showing bone tissue and bone marrow per section. ns: non significant.

**Figure 2:**
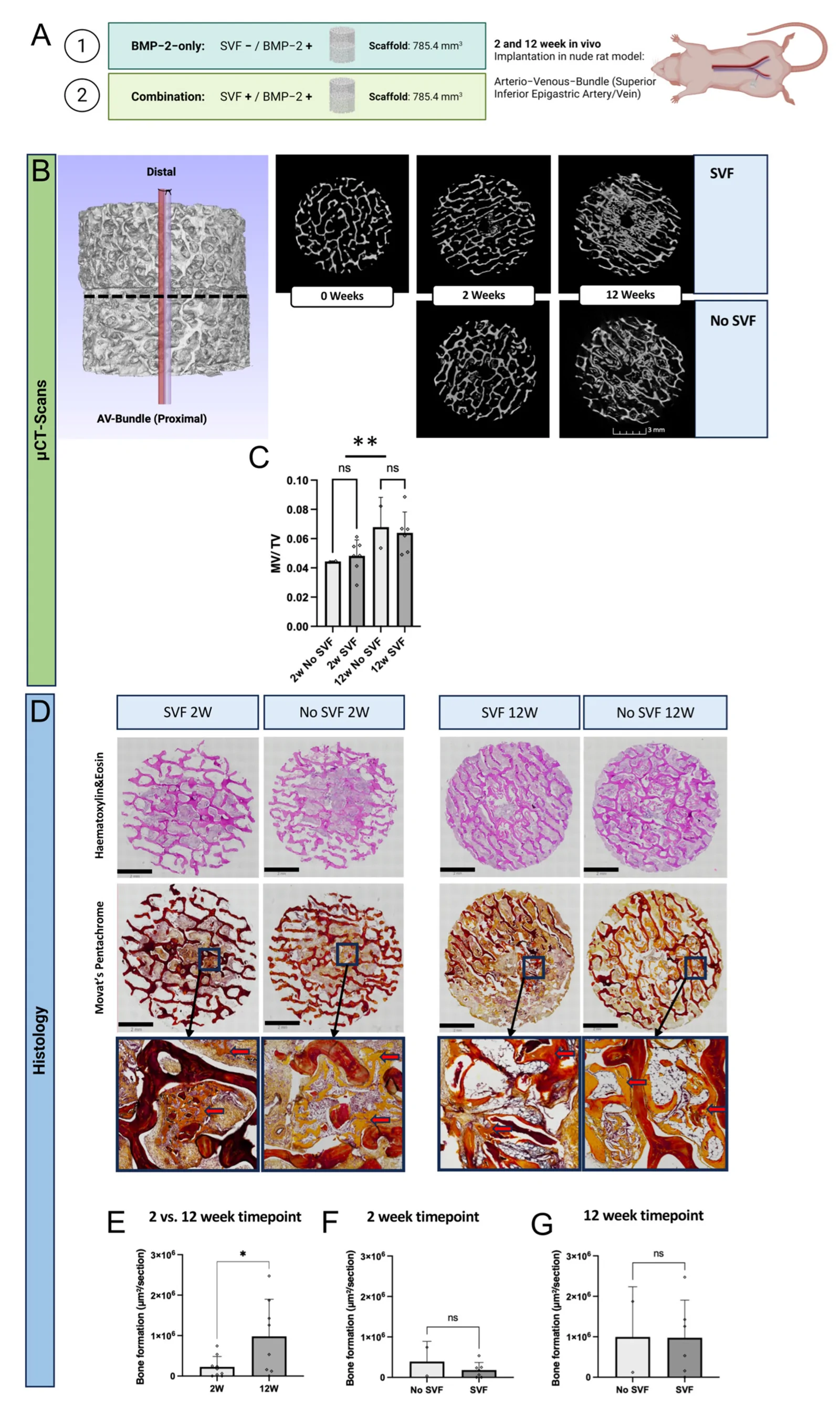
Bone formation and biofabrication in a nude rat model at 2 and 12 weeks. A total of 17 grafts were available for analysis: 9 at 2 weeks and 8 at 12 weeks. (**A**) Schematic of the experimental conditions. (**B**) X-ray tomographic pictures of the samples before and after explantation. (**C**–**E**) Quantification of mineralized volume (MV) per total volume (TV) at 2 and 12 weeks in samples containing SVF cells or not (**C**). (**D**) H&E and Movat’s Pentachrome staining of histological sections and insets showing higher magnification (scale bar = 2 mm). Red arrows show mineralized fibrous tissue at 2 weeks and bone tissue at 12 weeks. (**E**–**G**) Histomorphometric quantification of the section surface showing bone tissue and bone marrow per section at 2 and 12 weeks in all samples (**E**) and at 2 weeks (**F**) and 12 weeks (**G**) for samples containing SVF cells or not. ns: non significant, *: *p* < 0.05, **: *p* < 0.01.

**Figure 3:**
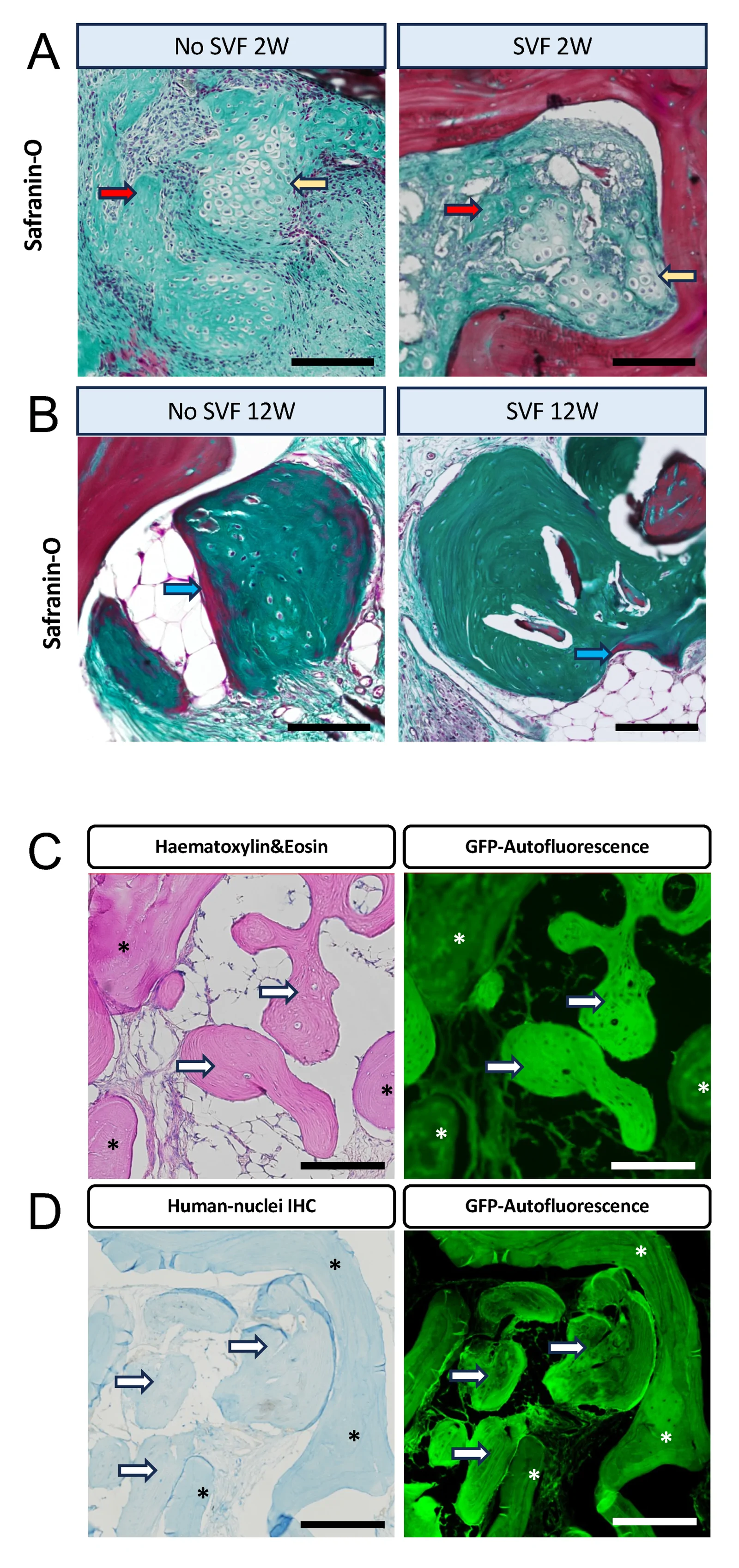
Analysis of bone tissue formation. (**A**,**B**) Safranin-O staining of histological sections after 2 weeks (**A**) or 12 weeks (**B**) in vivo, in the presence or absence of SVF cells. Scale bar = 100 μm. Red arrows show osteoid formation, while yellow arrows show cartilaginous tissue and blue arrows show mature bone formation, asterisks show the DBM. (**C**) H&E and GFP-channel fluorescence of bone tissue. Scale bar = 100 μm. White arrows show newly formed bone tissue, asterisks show the DBM. (**D**) Immunohistochemistry for human nuclei. Scale bar = 100 μm. White arrows show absence of human nuclei-positive cells in the newly formed bone at 12 weeks. Asterisks show the DBM.

## Data Availability

All relevant data are within the paper and its supporting information files.

## Supplementary Materials

The following supporting information can be downloaded at https://www.mdpi.com/article/10.3390/jfb17100520/s1, Figure S1: Comparison of bone formation in the two rodent models. Histomorphometric quantification of the section surface with bone tissue per total area in the subcutaneous and AV-bundle-based animal models. ns: non significant. Figure S2: Single occurrence of human cells in fibrous tissue after 2 weeks of implantation in the AV-bundle-based model. Immunohistochemistry for human nuclei (stained in brown). Diameter of the scaffold circle = 5 mm.
